# Hyperglycemic Myocardial Damage Is Mediated by Proinflammatory Cytokine: Macrophage Migration Inhibitory Factor

**DOI:** 10.1371/journal.pone.0016239

**Published:** 2011-01-25

**Authors:** Xi-Yong Yu, Hong-Mei Chen, Jia-Liang Liang, Qiu-Xiong Lin, Hong-Hong Tan, Yong-Heng Fu, Xiao-Ying Liu, Zhi-Xin Shan, Xiao-Hong Li, Hua-Zhang Yang, Min Yang, Yangxin Li, Shu-Guang Lin

**Affiliations:** 1 Medical Research Center, Guangdong Provincial Cardiovascular Institute, Guangdong General Hospital, Guangdong Academy of Medical Sciences, Guangzhou, People's Republic of China; 2 Division of Endocrinology, Guangdong General Hospital, Guangdong Academy of Medical Sciences, Guangzhou, People's Republic of China; 3 Texas Heart Institute and University of Texas Health Science Center, Houston, Texas, United States of America; University of Padova, Medical School, Italy

## Abstract

**Background:**

Diabetes has been regarded as an inflammatory condition which is associated with left ventricular diastolic dysfunction (LVDD). The purpose of this study was to examine the expression levels of macrophage migration inhibitory factor (MIF) and G protein-coupled receptor kinase 2 (GRK2) in patients with early diabetic cardiomyopathy, and to investigate the mechanisms involved in MIF expression and GRK2 activation.

**Methods:**

83 patients in the age range of 30-64 years with type 2 diabetes and 30 matched healthy men were recruited. Left ventricular diastolic function was evaluated by cardiac Doppler echocardiography. Plasma MIF levels were determined by ELISA. To confirm the clinical observation, we also studied MIF expression in prediabetic rats with impaired glucose tolerance (IGT) and relationship between MIF and GRK2 expression in H9C2 cardiomyoblasts exposed to high glucose.

**Results:**

Compared with healthy subjects, patients with diabetes have significantly increased levels of plasma MIF which was further increased in diabetic patients with Left ventricular diastolic dysfunction (LVDD). The increased plasma MIF levels in diabetic patients correlated with plasma glucose, glycosylated hemoglobin and urine albumin levels. We observed a significant number of TUNEL-positive cells in the myocardium of IGT-rats but not in the control rats. Moreover, we found higher MIF expression in the heart of IGT with cardiac dysfunction compared to that of the controls. In H9C2 cardiomyoblast cells, MIF and GRK2 expression was significantly increased in a glucose concentration-dependant manner. Furthermore, GRK2 expression was abolished by siRNA knockdown of MIF and by the inhibition of CXCR4 in H9C2 cells.

**Conclusions:**

Our findings indicate that hyperglycemia is a causal factor for increased levels of pro-inflammatory cytokine MIF which plays a role in the development of cardiomyopathy occurring in patients with type 2 diabetes. The elevated levels of MIF are associated with cardiac dysfunction in diabetic patients, and the MIF effects are mediated by GRK2.

## Introduction

Cardiomyopathy and cardiac dysfunction is a frequent complication in diabetic patients[1]. Diabetic patients develop a characteristic cardiomyopathy of ventricular hypertrophy and diastolic dysfunction which can be detected by cardiac tissue Doppler[2]. Poirior et al has shown that left ventricular diastolic dysfunction (LVDD) represent the first stage of diabetic cardiomyopathy[3]. Previous studies have demonstrated that the diastolic dysfunction and abnormal left ventricular mass can be ameliorated by tight glycemic control[4], indicating that hyperglycemia is a critical factor in diabetic cardiomyopathy. In the chronic state, high blood glucose induced by the effect of impaired glucose tolerance (IGT), especially postprandial hyperglycemia, may cause cardiac damage and dysfunction or apoptosis, eventually lead to the development of heart failure [5], [6].

It has been shown that a number of cytokine and inflammation factors are abnormally expressed in diabetic myocardium and contribute to myocardial fibrosis. Macrophage migration inhibitory factor (MIF) is one of the cytokines that has been shown to be increased in patients with type 2 diabetes. Importantly, MIF is associated with impaired glucose tolerance in type 2 diabetes[7]. MIF also induced the expression of matrix metalloproteinase (MMP9) which is involved in remodeling[8]. A recent report indicate that MIF mediates late cardiac dysfunction after burn injury[9]. Furthermore, MIF has been considered a cardiac-derived myocardial depressant factor[10]. Previous in vitro studies showing a role of MIF in inducing apoptosis in AC16 human cardiomyocytes exposed to high glucose levels[11]. However, the role of MIF in diabetic cardiomyopathy has not been studied in vivo and the signaling pathways mediating MIF's effect remains unknown.

Desensitization of G protein–coupled receptors (GPCR) is emerging as an important feature of several cardiovascular diseases. G protein–coupled receptor kinase 2 (GRK2) plays a key role in the regulation of a variety of these receptors, and its cardiac expression levels are altered in pathological situations such as chronic heart failure[12]. In the present study, we sought to compare the circulating MIF levels in diabetic patients with or without LVDD. Furthermore, we examined MIF expression in prediabetic rats and in H9C2 cardiomyoblasts cells exposed to high glucose level. In order to identify the downstream factors that mediate MIF's effect, we also studied the expression of GRK2 in H9C2 exposed to high glucose and MIF.

## Materials and Methods

### Patient study

This study recruited 83 type 2 diabetes patients (aged 30–64 years; forty-seven males and thirty-six females, without evidence of hypertension, coronary artery disease, congestive heart failure, diabetic complications; and with a maximal treadmill exercise test showing no ischemia), and 30 age-sex matched healthy adults. LV diastolic function was evaluated by cardiac tissue Doppler echocardiography, and systolic function was normal in all subjects. Pseudonormal pattern of ventricular filling and E'/A'<1 was regarded as LVDD. The diabetes subjects with normal left ventricle diastolic function were used as diabetic controls. All subjects had their fasting blood samples taken. The blood samples were centrifuged, and plasma were separated and frozen at −70°C. The clinical investigation was conducted according to the principles expressed in the Declaration of Helsinki, and approved by the Research Ethics Committee of Guangdong General Hospital (Guangzhou, China),the approval number was No. GDREC [2009]101. All subjects gave written consents to participate in this study, and were told to follow only conventional medical treatments without additional burden.

Plasma insulin concentrations were determined by ROCHE Elecsys 2010 (ROCHE Diagnostics GmbH). The sensitivity of this assay is 0.26 µU/ml, whereas the intraassay precision is 2.6% and interassay precision is 5.8%. Plasma glucose, triglyceride, LDL cholesterol, HDL cholesterol and total cholesterol levels were measured by Synchron systems LX20 (Beckman Coulter, Fullerton, CA). Glycated hemoglobin was determined using a commercial kit (Bio-Rad DiaSTAT analysor, BIO-RAD Laboratories Inc. Hercules, CA). The homeostasis model assessment of insulin resistance (HOMA-IR) was used as a substituted marker of insulin resistance. HOMA-IR was calculated using the following formula: HOMA-IR  =  fasting insulin (microunits per milliliter) × fasting glucose (micromoles per liter)/135.

### Animal Study

Sprague-Dawley (SD) rat were obtained at 6–8 wk of age from Laboratory Animal Center of Sun Yat-sen University and maintained in a specific pathogen-free environment. Commercial chow and tap water were made available ad libitum. All animals received a care in compliance with the Guide for the Care and Use of Laboratory Animals of US National Research Council in 1996, and the program was also approved by the research ethics committee of Guangdong General Hospital, the approval number was No. GDREC[2009]102(Animal). According to previous reported procedures [13], the rats were randomly assigned to a normal rat chow, or a high-fat diet. The normal mouse chow and high-fat diet contained, respectively, 3% and 20% fat, 20% and 18% protein, 45% and 33% carbohydrate. There was an adjunction of 5% salt in high-fat diet. For the IGT-rat, the tail-vein was injected with 20 mg/kg streptozotocin (STZ, Sigma, St. Louis, MO), and the drink-water was added by 0.04% N (G)-nitro-L- arginine methyl ester (L-NAME, Sigma, St. Louis, MO) and 10% fructose(Archer Daniels Midland Comp, Decatur IL). After 4 wk, all rats were anesthetized with 4-6.25 mg/kg Diazepam and 2–3.5 mg/kg Ketamine. Whole hearts were removed, snap frozen in liquid nitrogen, and stored at −80°C or fixed in 10% neutral-buffered formalin for 24 h and placed in 70% ethanol.

The fasting blood glucose (FBG) levels and glucose tolerance of the rats were detected at 4 wk. Briefly, animals were food-restricted overnight and subjected to oral glucose tolerance test (OGTT) using a glucose feeding (1 g/kg) by intragastric administration. Blood (∼0.25 ml) was collected from a cut at the end of the tail immediately at 0, 15, 30, 60, 90 and 150 minutes after glucose administration. and then the blood glucose (BG) was assayed by test strip (Roche, Accu-Check, Germany) and assessment of area-under-the-curve (AUC) for 150 min of BG concentration used the following formula[14]: AUC_BG_ = 7.5*BG0+15*BG15+22.5*BG30+30*BG60+45*BG90+30*BG150.

Cardiac function was measured at 4 wk. The rats were sedated with Ketamine, and left parasternal images were taken in the right lateral decubitus position with a 13-MHz transducer (Acuson, Mountain View, CA).

### Cell culture study

H9C2 cardiomyoblast (ATCC, Manassas, VA) were cultured with different concentrations of glucose (0, 5.5, 12.5, 25 and 33 mmol/L, adjusted the same osmotic pressure with manitol). Total RNA was isolated at 6 h after glucose treatment from the H9C2 cardiomyoblasts cell cultured in 6 wells plate using Trizol Reagent. In addition, 24 h after glucose treatment, the cells were split by cell disruption solution and incubated at −80°C for a further 24 h to adequately extract soluble proteins. After melting, the homogenates were disposed by ultrasonication, then centrifuged at 5,000 ×g, and the supernatant was collected and stored at −20°C until use.

AMD3100 (Sigma, St Louis, MO, USA) was used to inhibit CXCR4 on H9C2 cells. Because CXCR4 has been recognized as one part of MIF receptor complexes [15], this study was designed to investigate whether GRK2 expression induced by high glucose is mediated by MIF-CXCR4 pathway. First, H9C2 cells were pre-treated with different concentrations of AMD3100 (0, 1, 2.5, 5, 10 µg/mL) for 1 h, and then cultured in 25 mM glucose medium for 24 h. Second, H9C2 cells were pre-treated with 5 µg/mL AMD3100 for 1 h, and then cultured in 25 mM glucose medium for 3, 6, 12, 24, 48 h. The low glucose (5 mM) was used as the normal control. They were then analyzed by western blotting for GRK2 expression. All experiments were repeated at least three times.

### Determination of plasma MIF

Plasma MIF was assayed by a sandwich enzyme-linked immunosorbent assay using an antibody pair and recombinant MIF from R&D Systems (Wiesbaden, Germany). The intra-assay precision for this assay is 3.5%, and the interassay precision is 12%. The calculated sensitivity of this assay is 5 pg/ml, and the lowest standard concentration measured in this assay was 30 pg/ml. The mean for normal range for healthy subjects was 1.2±0.6 ng/ml (range: 0–2.3 ng/ml). There was no cross-reactivity with seven recombinant proteins tested (macrophage chemoatractant protein-1, TNF, matrix metalloproteinase-9, interleukin-4, interferon-γ, tissue factor, and soluble intercellular adhesion molecule-1). All samples were assayed together in two large batches.

### Determination of MIF and GRK2 mRNA level in circulatory lymphocytes

Total RNA was isolated from peripheral lymphocytes which were separated from peripheral blood using Trizol Reagent. In brief, the real-time PCR assay includes two steps. Reverse transcription was performed by using ThermoScript^TM^ RT-PCR system. PCR conditions were 95°C for 3 min, followed by 34 cycles of 94°C for 50 s, 58°C for 30 s and 72°C 45 s for MIF, and 36 cycles of 94°C for 50 s, 58°C for 40 s and 72°C 50 s for GRK2 in the Bio-Rad MJ OPTICON-II apparatus. Fluorescence changes were monitored with SYBR Green PCR Supermix (Bio-Rad) after every cycle, and melting curve analysis was performed at the end of 35 cycles to verify PCR product identity. Each PCR reaction was repeated three times, and the average median threshold cycle values were used for analysis. To normalize RNA content, the GAPDH was used as an internal control. The PCR primers were as follow: GAPDH: sense: 5′-GTG GGC CAT GAG GTC CAC-3′; antisense: 5′-TCC ATG ACA ACT TTG GTA TCG T-3′ (478 bp); MIF: sense: 5′-TCA CCG CCT CGG CTT GTC A-3′; antisense: 5′-ATG AAC TTT CTG CTG TCT TG-3′ (198 bp); GRK2: sense:5′-TTC TCG AAG AGT GCC ACT G-3′, antisense: 5′-CAT TCA TGG TCA GGT GGA TG-3′ (202 bp).

### Western blot assay

To measure the protein levels of MIF and GRK2 in H9c2 cardiomyoblasts cells treated with glucose, Protein was extracted from cultured cells with ice-cold lysis buffer consisting of 50 mmol/L HEPES (pH 7.4), 1% Triton-X100, 2 mmol/L sodium orthovanadate, 100 mmol/L sodium fluoride, 1 mmol/L edetic acid (EDTA), 1 mmol/L phenylmethylsulphonyl fluoride (PMSF), 10 mg/L aprotinin and 10 mg/L leupetin (Sigma, St Louis, MO, USA). After centrifugation at 12000×g centrifugation for 5 min, the protein content of the supernatant was determined using the Quick Start™ Bradford assay (Bio-Rad, Hercules, CA). Then, cellular protein extracts were separated by gradient polyacrylamide gel electrophoresis (12–15%) and transferred to polyvinylidene difluoride membranes (PVDF membranes). The membranes was probed with primary polyclonal rabbit anti-MIF, polyclonal rabbit anti-GRK2 and polyclonal goat anti-GAPDH (Santa Cruz, CA) according to the procedures recommended by the manufacturers, followed by a goat anti-rabbit IgG–horse radish peroxidase and mouse anti- goat IgG– horse radish peroxidase, respectively. Immune reactivity was visualized with ECL plus Western Blotting Detection reagents (Amersham Biosciences, Buckinghamshire, UK) using procedures recommended by the manufacturer, then the signals were quantified by densitometry.

### Construction of MIF siRNAs plasmid

Three candidate siRNAs targeting MIF gene were selected by using the siRNA designing programme of the Ambion (Ambion Inc, Austin, TX). The DNA templates for MIF siRNAs were annealed by two complimentary oligonucleotide strands. The loop sequence of the two DNA templates was TTCAAGAGA, with the complimentary sequences at the bilateralis and two sticky end sequences corresponding to the terminations of the linearized pSilencer-4.1-neo. According to the targeting sequence of the MIF siRNA, CAGGGTCTACATCAACTATTA, two single oligonucleotide strands were designed and synthesized. The sequence of the sense strand was 5′-GAT CCG GGT CTA CAT CAA CTA TTA TTC AAG AGA TAA TAG TTG ATG TAG ACC CTG A-3′, and the antisense strand was 5′-AGC TTC AGG GTC TAC ATC AAC TAT TAT CTC TTG AAT AAT AGT TGA TGT AGA CCC G-3′.

### Plasmid transfection and siRNA Interference

Before the start of the experiment, 3×10^5^ H9C2 cells were inoculated in 6-well plates. After the cell abundance reached 60% to 70%, the MIF-siRNA plasmid was transfected into the cells by using Lipofectamine 2000™ reagent (Invitrogen, Carlsbad CA, USA). A negative siRNA plasmid was used to control. In order to observe the specificity, one of the transfected groups was treated with 50 ng/mL rhMIF to neutralize the siRNA activity. The recombinant human MIF (rhMIF) was cloned, expressed in Escherichia coli, and purified from the soluble fraction of the cell lysate as previous study[8]. This rhMIF contained <10 pg of endotoxin per microgram of recombinant protein, as determined by the chromogenic Limulus amebocyte assay (Chromogenix, Sweden).

### Glycogen staining

Briefly, after deparaffinization and rehydration, the sections of heart tissues were stained with periodic acid-Schiff's reagent (PAS, Polyscientific, Bay Shore, NY) for glycogen and finally counterstained with hematoxylin, according to the manufacturer's guidelines.

### TUNEL staining

Heart tissues were fixed in 10% formalin, embedded in paraffin, and sectioned at 5 µm. And in situ TUNEL detection kit from Keygen (Nanjing, China) was used according to the manufacturer's instructions. Briefly, after deparaffinization and rehydration, the sections were treated with H_2_O_2_ and incubated with the reaction mixture containing TdT and digoxigenin-conjugated dUTP for 1 h at 37°C. Labeled DNA was visualized with peroxidase-conjugated anti-digoxigenin antibody using 3,3′-diaminobenzidine (DAB) as the chromogen. For negative control, TdT was omitted from the reaction mixture.

### Statistical analysis

Statistical analysis was carried out using SPSS13.0 software. Continuous data are expressed as the mean ± SD. Statistical analysis was carried out using unpaired t test between diabetes controls and diabetes with LVDD subjects. Correlation analysis was performed using Spearman rank order correlation among plasma MIF, glucose level, and HOMA-IR, and body mass index. Results in vitro were analysis of variance (ANOVA) followed by student's-t-test. A p value of less than 0.05 was considered significant.

## Results

### Clinical characteristic of diabetic patients with LVDD

We found increased levels of blood glucose and triglyceride in diabetic patients who also developed insulin resistance (HOMA-IR). Higher levels of glycosylated hemoglobin, and albuminuria were found in diabetic patients with LVDD compared to diabetic patients without LVDD (Table 1). We found no significant differences between the two groups of patients on parameters such as age, body mass index, disease period, HOMA-IR and total cholesterol.

**Table 1:** Clinical features of study subjects.

	*Normal(n = 30)*	*DM (n = 46)*	*DM+LVDD(n = 37)*
Age(years)	40 (30–55)	48 (30–62)	51(37–64)
Disease period (months)	0	48 (11–99)	52 (14–166)
Body mass index (kg/m^2^)	22.85±1.87	23.89±0.76	23.58±5.64
Fasting blood glucose (mmol/L)	4.77±2.81	10.25±3.92**	9.66±3.34**
Postglandial blood glucose (mmol/L)	6.06±2.39	13.58±4.72**	14.87±5.66**
Glycosylated hemoglobin (%)	6.19±1.55	8.54±2.21*	9.56±1.64* #
HOMA-IR	1.22±0.67	1.79±0.95*	1.84±1.02*
Total cholesterol (mmol/L)	4.68±0.79	4.75±0.85	4.71±0.91
Triglyceride (mmol/L)	1.18±1.11	1.98±1.66*	2.05±1.91*
LDL (mmol/L)	2.23±1.08	2.43±0.97	2.55±0.70
HDL (mmol/L)	1.24±0.97	1.11±0.68	1.19±0.69
Urine albumin concentration (mg/mmol.Cr)	10.69±7.44	17.14±16.28*	35.18±34.09** #

1 Results are presented as mean ±SD, compared with Normal,

*p<0.05,

**p<0.01; Compared with DM,

#*p<0.05*.

2 Normal: healthy subjects; DM: type 2 diabetes mellitus patients; DM+LVDD: DM patients with left ventricular diastolic dysfunction. The urine albumin concentration is the ratio to creatinine (Cr).

Left ventricular diastolic function was evaluated by tissue Doppler echocardiography. The ratio of the mitral blood flow velocity during early diastole to atrial contraction (E/A) in diabetes with LVDD decreased significantly compared to healthy subjects or diabetic patients without LVDD (0.67±0.34 vs 1.46±0.44 or 1.37±0.42, p<0.01). We also observed reduced ejection fraction (EF) in diabetic patients with LVDD compared to healthy subjects or in diabetic patients without LVDD (46.0±7.8 vs 61.55±8.07 or 60.8±9.7, p<0.01) (Table 2).

**Table 2:** The evaluation of left ventricular function in study subjects.

	*Normal* *(n = 30)*	*DM* *(n = 46)*	*DM+LVDD* *(n = 37)*
E/A ratio	1.46±0.44	1.37±0.42	0.67±0.34** ##
LVIDs (cm)	28.96±3.61	25.13±3.81*	25.92±4.26*
LVIDd(cm)	46.32±4.02	45.47±3.13	41.33±3.57* #
LVSD(cm)	7.89±0.78	9.47±0.99	10.45±1.35* #
LVPWD(cm)	7.71±0.95	8.01±0.81	8.57±1.18*
EF(%)	61.55±8.07	60.8±9.7	46.0±7.8** ##

1 Compared to Normal,

*p<0.05,

**p<0.01; Compared to DM,

#p<0.05,

##p<0.01.

2 E/A ratio: the mitral blood flow velocity during early diastole (E)/atrial contraction (A); LVIDs: left ventricular internal diameter at end-systole; LVIDd: left ventricular internal diameter at end-diastole; IVSD: interventricular septum thickness at end-diastole; LVPWD: left ventricular posterior wall thickness at end-diastole; EF: ejection fraction.

### MIF levels are altered in diabetic patients with LVDD

**Figure 1:**
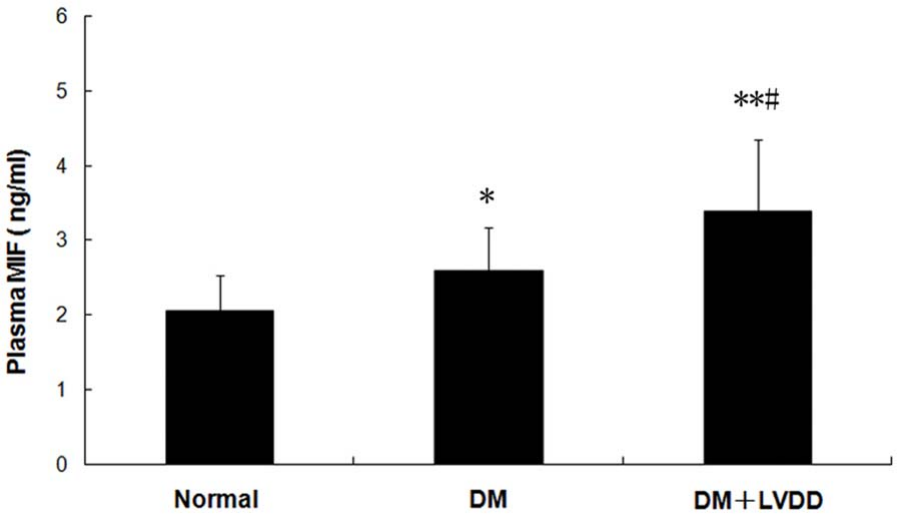
The levels of plasma MIF in type 2 diabetic patients with or without LVDD. Results are presented as mean ± SD, compared with health control, *p<0.05, ** p<0.01; compared with diabetic patients without LVDD, *#p<0.05*.

We found higher concentrations of MIF in the plasma of DM subjects compared to healthy subjects (DM with LVDD 3.39±0.96, without LVDD 2.59±0.59 vs. Normal 2.05±0.49 ng/ml, p<0.05). The plasma MIF concentrations of patients with type 2 diabetes with LVDD were significantly higher than that of diabetic patients without LVDD (3.39±0.96 vs 2.59±0.59 ng/ml, p<0.05) (Figure 1).

**Figure 2:**
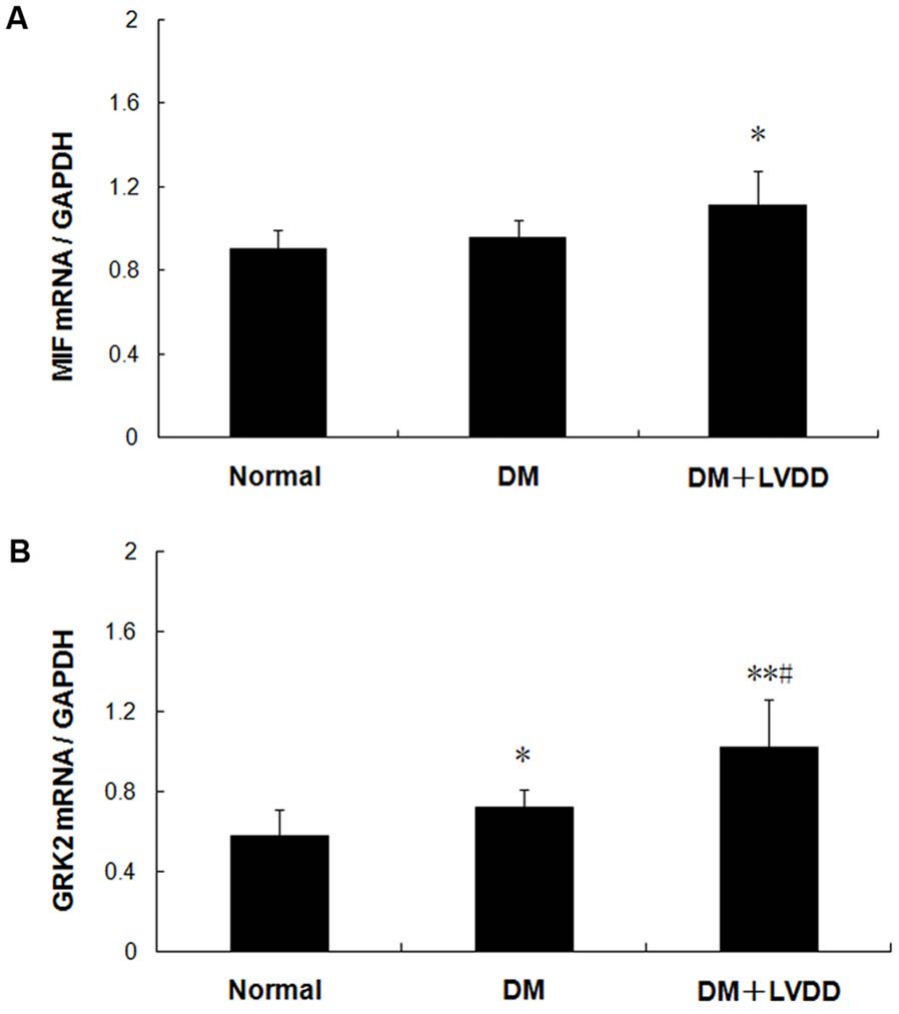
The levels of lymphocyte MIF mRNA (A) and GRK2 (B) in type 2 diabetic patients with or without LVDD. Results are presented as mean ± SD, compared with health control,*p<0.05, ** p<0.01; compared with diabetic patients without LVDD, *#p<0.05*.

**Figure 3:**
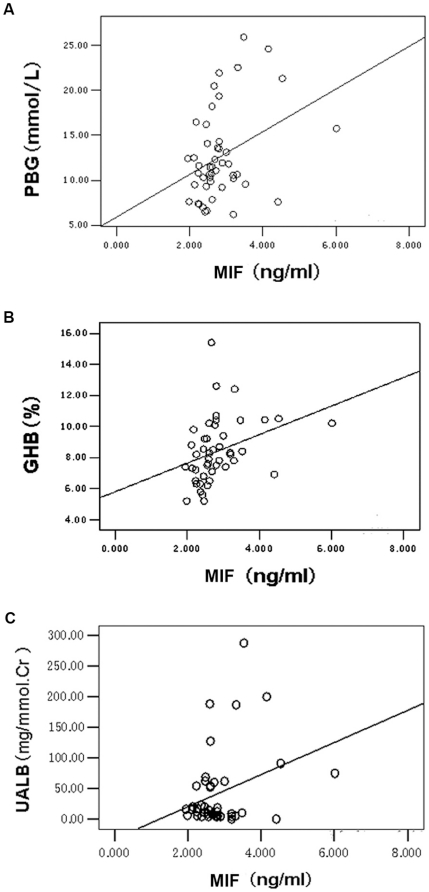
Correlations of postglandial blood glucose (PBG), glycosylated hemoglobin (GHB) and urine albumin (uALB) levels with plasma MIF respectively in diabetic patients with LVDD. The statistical analysis indicated that PBG, GHB and uALB level correlated with plasma MIF in diabetic patients with LVDD (r = 0.354, *p<0.05*; r = 0.336, *p<0.05*; r = 0.312, *p<0.05*, respectively).

Real-time quantitative RT-PCR assay revealed that the level of MIF mRNA expression in peripheral lymphocytes from the diabetic patients with LVDD was significantly higher than that of diabetic patients without LVDD (Figure 2A) and mRNA expression of GRK2 followed a similar pattern as MIF (Figure 2B). Furthermore, postglandial blood glucose (PBG), glycosylated hemoglobin (GHB) and urine albumin (uALB) level correlated with plasma MIF in diabetic patients with LVDD (r = 0.354, p<0.05; r = 0.336, p<0.05; r = 0.312, p<0.05, respectively) (Figure 3).

### FBG levels and MIF levels are increased in IGT-rats

FBG levels of IGT-rats were higher than those of controls at 4 wk (Table 3). The glucose metabolism of rats was evaluated by OGTT. We found that the AUC_BG_ of IGT-rats was higher than that of controls (p<0.05). The data indicate that the glucose metabolism of IGT-rats was imbalanced such that glucose couldn't be utilized or metabolized normally in many organs, including heart. Furthermore the levels of MIF and CRP were also higher in IGT rats than that of the controls (p<0.05).

**Table 3:** Comparison of biomarkers in control and IGT rats (mean±SD).

	Controls (n = 6)	IGT rats (n = 6)
FBG (mmol/L)	4.26±1.34	5.37±1.49*
AUC_BG_ (mol/L•150 min)	1.14±0.37	1.58±0.29*
CK-MB (U/L)	493.9±186.1	2682.4±894.9**
NT-proBNP (pg/ml)	65.6±10.5	514.3±204.1**
CRP (mmol/L)	2.72±1.87	4.97±1.99**
IGF-1 (pg/ml)	4068.3±311.3	2991.5±401.5*
MIF (ng/ml)	24.8±4.0	32.5±4.7*

*p<0.05,

**p<0.01 *vs.* controls.

FBG: Fasting blood glucose; AUC_BG_: area-under-the-curve of blood glucose in oral glucose tolerance test; CK-MB: MB isoenzyme of creatine kinase; NT-proBNP: N-terminal pro-brain natriuretic peptide; CRP: C-reactive protein; IGF-1: insulin-like growth factor-1; MIF: macrophage migration inhibitory factor; IGT: impaired glucose tolerance.

### Echocardiography assay for the cardiac function in IGT-rats

Cardiac function in IGT-rats and controls was assessed by echocardiography. After 4 wk, the EF and LVFS in IGT-rats were much lower than that of the controls, indicating that the cardiac diastole and construction were impaired in IGT-rats. LVDd, LVDs and PWd were also abnormal in IGT-rats, suggesting the ongoing cardiac remodeling in IGT-rats. (Table 4).

**Table 4:** Echocardiographic assessment of cardiac function in rats (mean±SD).

	Controls (n = 6)	IGT rats (n = 6)
LVDd (mm)	4.21±0.21	5.68±0.45*
LVDs (mm)	2.43±0.08	3.74±0.33*
EF (%)	81.60±1.96	69.32±2.83*
LVFS (%)	44.78±1.87	34.33±2.08*
HR (beats/min)	449.24±16.21	428.16±8.70*
PWd (mm)	2.94±0.44	1.88±0.18*

*p<0.05 *vs.* controls.

LVDd: left ventricular end diastolic dimension; LVDs: left ventricular end-systolic dimension; EF: ejection fraction; LVFS: Left ventricular fractional shortening; HR: heart rate; PWd: posterior wall diastolic; PWs: posterior wall systolic; IVSd: interventricular septum in diastole; IVSs: interventricular septum in systole; IGT: impaired glucose tolerance.

### The myocardium of IGT-rats is characterized by increased apoptosis and MIF expression

**Figure 4:**
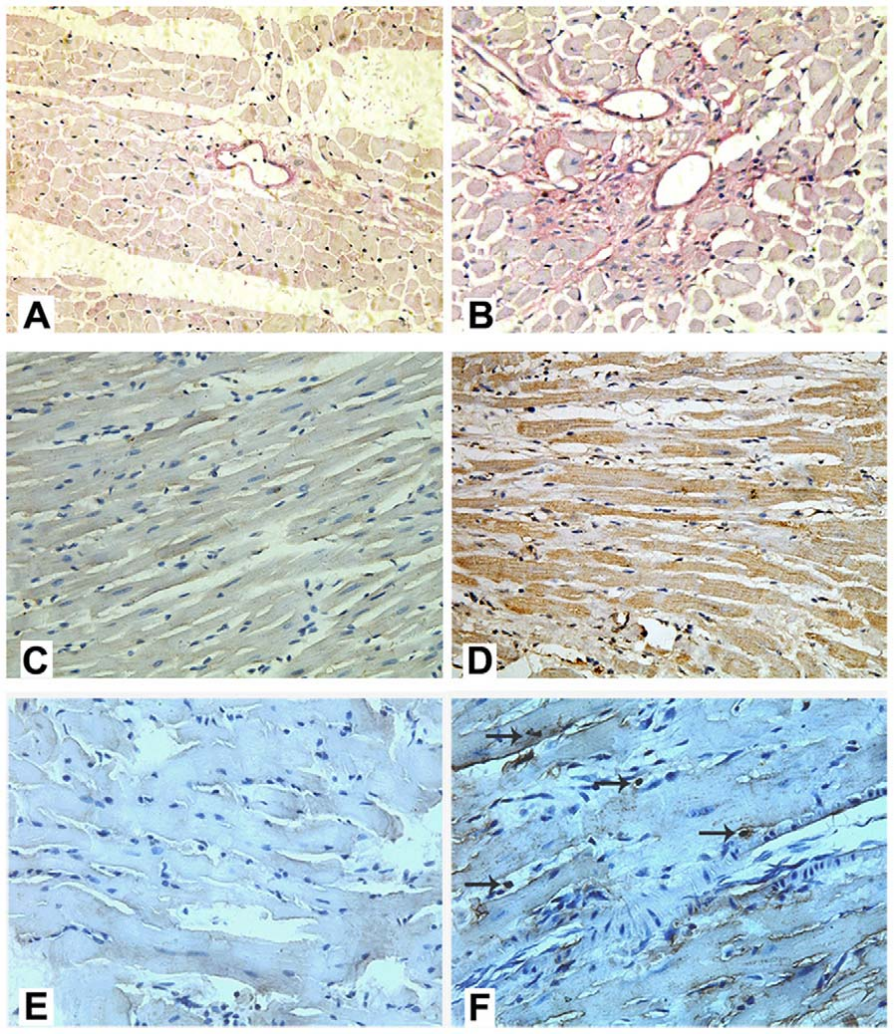
Comparison of myocardial pathology between control (A, C, E) and IGT (B, D, F) rats. A—B: Cardiac glycogen identified by PAS staining, the glycogen granules were stained into prunosus, cellular nuclei were stained into blue; C—D: macrophage migration inhibitory factor (MIF) detected by immunohistochemistry, the MIF was stained into brown; E—F: The apoptosis of myocardial cells detected by TUNEL, the brown showed apoptotic cells, the blue showed nuclei.

**Figure 5:**
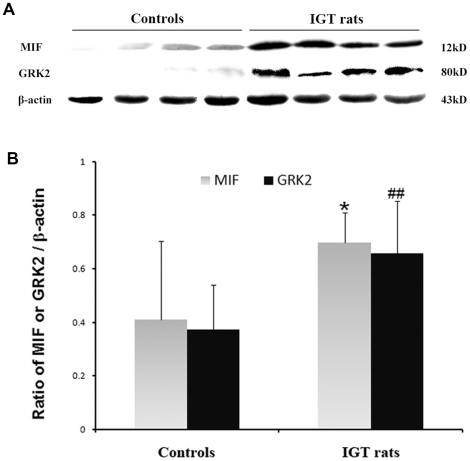
Western blot analysis of MIF and GRK2 protein expressions in the rat hearts. A: Representative western blots of heart lysates showing the expression of MIF and GRK2. B: Quantitative detection by densitometric analysis and normalized to β-actin (mean±SD, n = 4). Compared with control:* p<0.05, **p<0.01.

We found increased storage of glycogen in the heart of IGT-rat compared to that of the control animals (Figure 4A and 4B). Glycogen was mainly stored in the cardiac perivascular tissues of IGT-rat. MIF expression is higher in the heart of IGT with cardiac dysfunction compared to that of the controls (Figure 4C and 4D). We also observed a significant number of TUNEL-positive cells (45%±7%) in the myocardium of IGT-rats but not in the control rats. (Figure 4E and 4F). Western blot analysis showed higher expressions of MIF and GRK2 in IGT-rats than that in controls (Figure 5).

High glucose induced GRK2 expression is mediated by MIF via CXCR4 pathway.

**Figure 6:**
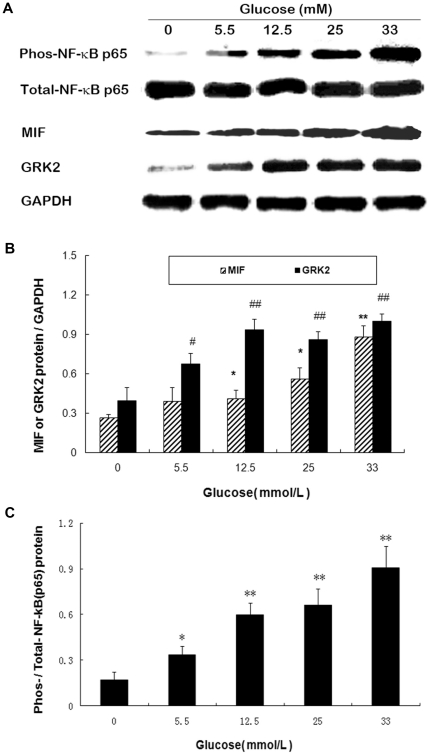
Western blot analysis of MIF and GRK2 expression, and the phosphorylation levels of NF-κB in H9C2 cells. H9C2 cardiomyoblasts were treated with various concentrations of glucose (0, 5.5, 12.5, 25 and 33 mmol/L) for 24 hours. A: Representative blot map; B: Quantitative analysis of MIF and GRK2 expression; C: Quantitative analysis of the ratio of phosphorylated NF-κB.

**Figure 7:**
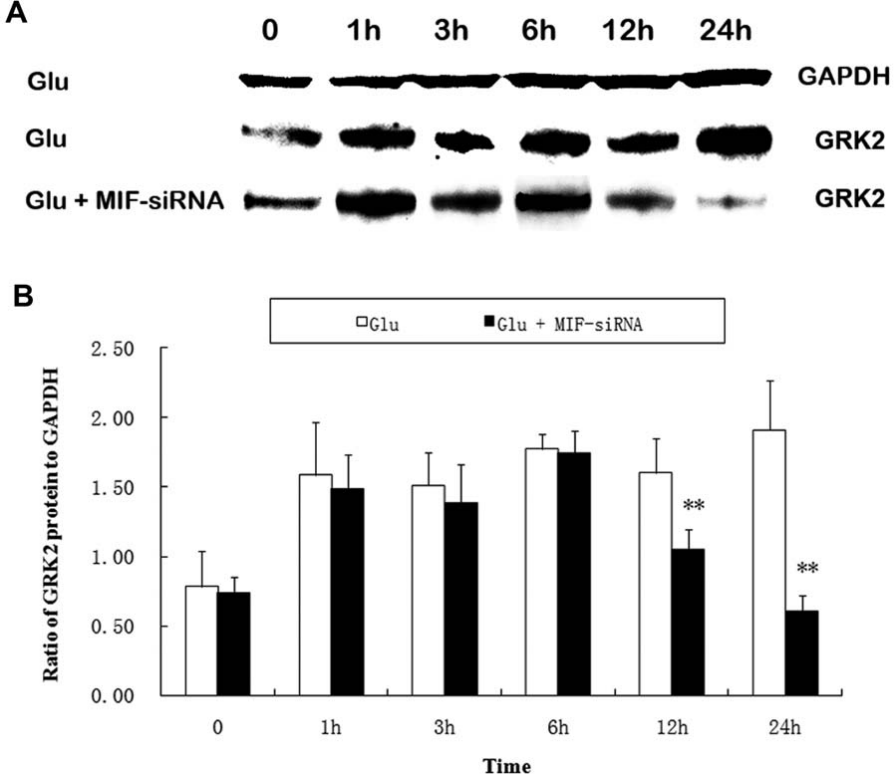
The effect of MIF knockdown on glucose-induced GRK2 expression. H9C2 cells were incubated with glucose (25 mmol/L) for different time. The cells were treated with either glucose, or combination with MIF siRNA. GRK2 expression was assessed by Western blot analysis. A: Representative blot map; B: Quantitative analysis of GRK2 expression.

Using Western blot, we examined the protein levels of MIF and GRK2 in H9C2 cardiomyoblasts with high glucose exposure. Our data showed glucose induced the expression of both MIF and GRK2 in a dose dependent pattern (Figure 6). To find out whether glucose induced GRK2 expression is mediated by MIF, MIF silencing was performed 12 hours before glucose treatment. We found that glucose-induced GRK2 expression was abolished by MIF siRNA (Figure 7). These data indicate that glucose induced GRK2 expression is mediated by MIF. Furthermore, MIF expression induced by high glucose was accompanied by the phosphorylation of NF-κB (Figure 6), suggested that NF-κB pathway may be involved in high glucose induced MIF expression.

**Figure 8:**
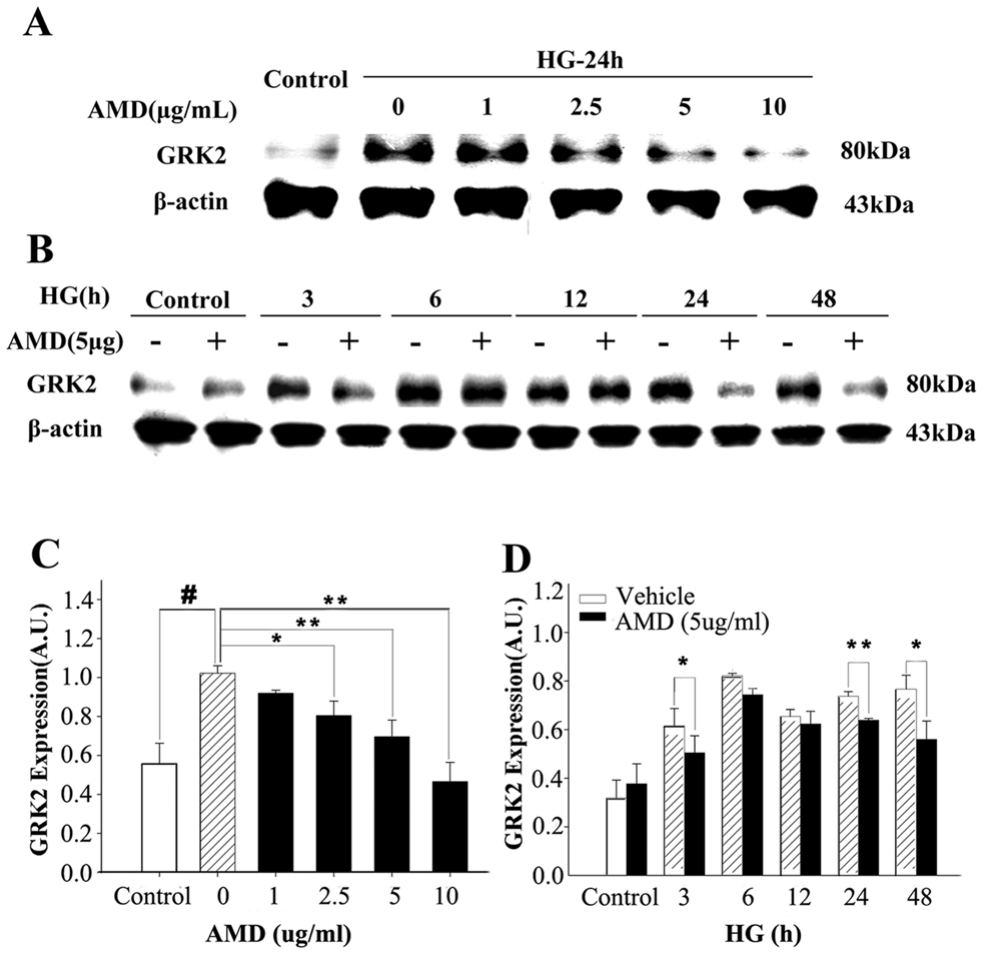
CXCR4 inhibitor attenuates GRK2 expression induced by high glucose. A: Representative western blots of cardiomyocyte lysates showing the effect of different concentrations of AMD3100 on GRK2 expression. B: Representative western blots of cardiomyocyte lysates showing the effect of AMD3100 on GRK2 expression at different time. C and D: Quantitative detection of GRK2 expression by densitometric analysis and normalization to β-actin. C: Compared with the high glucose group: # p<0.01, * p<0.05, ** p<0.01; D: Compared with vehicle: * p<0.05, ** p<0.01. (mean ± SD, n = 3).

To determine whether CXCR4 pathway is involved in MIF signaling and subsequent GRK2 expression, we treated H9C2 cells with high glucose in the presence or absence of CXCR4 inhibitor AMD3100. Compared with untreated cells, GRK2 protein expression increased at 24 h after high glucose treatment, but this increased GRK2 expression was abolished by various concentrations of CXCR4 inhibitor AMD3100, especially by 5 and 10 µg/mL (Figure 8A and C). AMD3100 has no effects on GRK2 expression in H9C2 cells in normal condition, but AMD3100 pretreatment prevented high glucose induced GRK2 (Figures 8B and D).

## Discussion

The major findings of the present study are that plasma levels of MIF is increased in diabetic patients with LVDD and MIF levels correlate with that of postglandial blood glucose, glycosylated hemoglobin and urine albumin. We demonstrated that both MIF and GRK2 mRNA levels are increased in circulatory lymphocytes from diabetic patient with LVDD. Our data indicate that MIF and GRK2 expression can be induced in H9C2 cells when exposed to high glucose levels. We further showed that the expression of GRK2 is induced by MIF. To our knowledge, this is the first report that GRK2 expression is upregulated in diabetic patients with LVDD and that GRK2 is regulated by MIF.

MIF is a pleiotropic cytokine that plays a crucial role in innate immune immunity as well as stress responses [16]. MIF is also associated with insulin resistance[17]. It has been shown previously that systemic concentrations of MIF are elevated in patients with obesity, impaired glucose tolerance and type 2 diabetes[18]. Recent studies also suggested a direct link between MIF and cardiovascular disease[19]. MIF is produced abundantly by various cells in all types of human atherosclerotic lesions[20], [21]. Genetic deletion and antibody inhibition studies established a causal link between MIF and atherosclerosis [22]. These previous reports are in line with our findings that MIF may be a causal factor of left ventricular diastolic dysfunction in patients with type 2 diabetes.

The mechanism through which MIF exerts its atherogenic properties may involve chemokine-like functions. It has been reported that MIF activates both chemokine receptors CXCR2 and CXCR4 and thereby mediates the recruitment of inflammatory cells like monocytes and lymphocytes [15], [23]. In the present study, we demonstrate that MIF could also exert its effect via the upregulation of GRK2, and the elevated GRK2 levels can be blocked by specific CXCR4 inhibitor, AMD3100. Therefore, over-expression of GRK2 induced by high glucose may be mediated by MIF-CXCR4 signaling pathway.

Heart failure is characterized by abnormalities at multiple levels in 7TM receptor signaling pathways such as chronic receptor desensitization of β-adrenergic receptors resulting in reduced receptor responsiveness. Because the expression and activity of GRK2 are increased in multiple animal models of heart disease and in human heart failure, it has been suggested that enhanced GRK2 activity can explain the concomitantly decreased receptor responsiveness[24]. GRK2 is a family of serine/threonine kinases that specifically phosphorylate agonist-occupied GPCRs[25]. The phosphorylation event leads to the uncoupling of beta-adrenergic and angiotensin receptors from their associated G protein and initiate the sequestration of the receptors.

In order to investigate the pathogenesis of cardiac dysfunction induced by IGT, we studied the cardiac glucose metabolism, apoptosis and expressions of MIF and GRK2 in animals. Our data showed that more glucose was converted to glycogen in the myocardial tissues of IGT rats than that in controls. These data suggest that in IDT rat, high glucose has modified myocardial energy metabolism and resulted in augmented fatty acid and decreased glucose consumption [26]. Consequently, superfluous or extra glucose that myocardium can't utilize is transformed into glycogen which is stored in hearts, resulting cardiac dysfunction [27]. Interestingly, perivascular myocardium of IGT rat was a main place for glycogen deposit, and it was inferred that the high blood glucose might first damage the areas close to blood vessels. Our data further showed that cardiac apoptosis in prediabetes state may lead to cell death and removal, thereby contributing to the cardiac function damage. Furthermore, we observed that the expression level of GRK2 was higher in the IGT rats than in controls. Our findings are in line with previous reports that abnormal up-regulation of GRK2 can chronically and constantly activate β1-adrenergic receptor (β1AR) and accelerate heart failure development [28].

Previous studies have shown that MIF may be induced by oxidative stress [29]. We and others have demonstrated that high glucose is a strong oxidative stress and apoptosis inducing factor [30]. It has been shown that MIF expression can be induced by oxidative stress through a NF-κB mediated mechanism [29], [31]. The cardiac dysfunction resulting from high glucose may be mediated partly by MIF, and NF-κB signaling pathway may be involved in this process [31]. In addition, the insulin resistance and type-2 diabetes were characterized by an increase in lipolysis and FFA concentrations, and elevated FFA levels also induced an increase in MIF [32]. Therefore, IGT caused a lot of disorder which come together to cause chronic inflammation, especially increase MIF, and finally result in myocardial damage.

In conclusion, our data suggest that MIF are involved in the pathogenesis of LVDD in patients with type 2 diabetes and GRK2 may play a role in mediating MIF's effect. These findings provided new insights into the role of inflammation in the development of diabetic cardiomyopathy which has a potential therapeutic implication.
